# Caspase-8: Friend or Foe in Bortezomib/Lenalidomide-Based Therapy for Myeloma

**DOI:** 10.3389/fonc.2022.861709

**Published:** 2022-03-07

**Authors:** Liang Zhou

**Affiliations:** Department of Pharmacology, College of Pharmaceutical Sciences, Soochow University, Suzhou, China

**Keywords:** caspase-8, bortezomib, lenalidomide, myeloma cell, proliferation, apoptosis

## Abstract

Antiproliferation and proapoptosis are two major molecular mechanisms of action of drugs used for the treatment of multiple myeloma. Proteasome inhibitors, such as bortezomib (PS-341), and immunomodulatory drugs (IMiDs), such as lenalidomide, are the two drug types approved for the treatment of myeloma. Bortezomib and lenalidomide activate caspase-8 and promote the apoptosis of myeloma cells. However, caspase-8 inhibition potentiated the antiproliferative effect of lenalidomide and bortezomib in myeloma cells, suggesting that caspase-8 could regulate proliferation and apoptosis in the opposite pathway. In this mini-review, I summarized recent advances in determining the molecular mechanisms of caspase-8 in bortezomib–lenalidomide-based therapy for myeloma and explored the possible functions of caspase-8 in the proliferation and apoptosis of myeloma cells. Furthermore, future directions of caspase-8-based therapy for myeloma have been discussed.

## Introduction

Caspase-8, as an initiator caspase, is essential for death receptor-dependent apoptosis (1, 2) and is activated in multiple pharmacological treatments for myeloma (3, 4). However, we have previously shown that caspase-8 activation attenuates the anti-myeloma effect of bortezomib and lenalidomide (5, 6).

Thalidomide and its analogs lenalidomide and pomalidomide directly bind to the cereblon (CRBN) (7) and subsequently recruit neo-substrates IKZF1/3 to the CRL4^CRBN^ E3 ligase, thereby inducing ubiquitination and degradation of IKZF1/3 and exhibiting an anti-myeloma effect (8, 9). The indispensability of CRBN for the anti-myeloma effect (10, 11) indicates that upregulation of CRBN can potentiate the anti-myeloma effect of lenalidomide (12–14). Recently, we discovered that the proteasome inhibitors bortezomib and MG-132 could induce CRBN cleavage, which possibly attenuates the anti-myeloma effect of lenalidomide (5, 6). However, combination therapy with lenalidomide and bortezomib is the first-line pharmacotherapy for multiple myeloma (15, 16) and has led to the paradoxical therapeutic mechanisms that mediate the action of lenalidomide and bortezomib in myeloma (17).

In myeloma cells, bortezomib inhibits the functioning of the 26S proteasome and affects multiple molecular pathways, including oxidative stress, NF-κB, and DNA damage and repair pathways, as well as classical intrinsic (mitochondria-dependent) and extrinsic (death receptor-dependent) cell death cascades, inducing the apoptosis of myeloma cells (4, 18–20). Lenalidomide exhibits an anti-myeloma effect by increasing apoptosis and inhibiting the proliferation of myeloma cells (3, 21, 22).

In this minireview, the caspase-8-involved extrinsic cell death cascade in bortezomib and lenalidomide therapy for myeloma was evaluated. First, I introduce the caspase-8 signaling pathway in myeloma treatment. Second, I summarize the recent advances in bortezomib and lenalidomide treatment in multiple myeloma and survey the different biological roles of caspase-8 in the treatment of myeloma. Finally, I discuss future perspectives on caspase-8-based therapy for myeloma.via cereblon IKZF1/3 cascade.

## Caspase-8 and “Programmed” Cell Death

**Figure 1:**
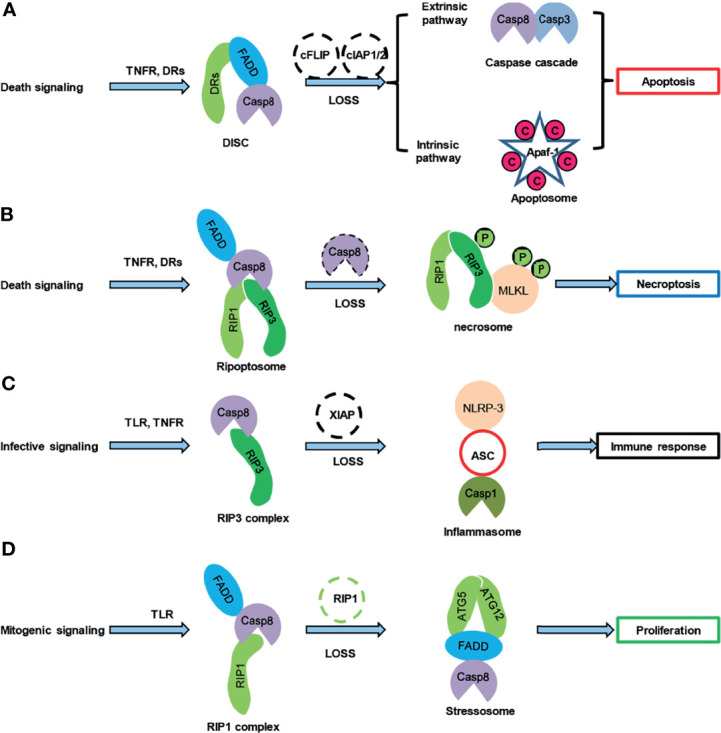
Caspase-8 was involved in multiprotein platforms. **(A)** Caspase-8 regulated the exterior and interior pathway-mediated apoptosis after the death-signaling challenge. **(B)** The loss of caspase-8 resulted in necroptosis. **(C)** Caspase-8 cleaved RIP3 and subsequently regulates the NLRP-3 inflammasome, thereby modulating the immune response after the infective signaling challenge. **(D)** Caspase-8 controlled the proliferation of lymphoid cells by interacting with the ATG5-ATG12 platform. C, cytochrome C; P, phosphorylation.

The Fas ligand (FasL, CD95 L) and tumor necrosis factor (TNF)-α bind to the relative cell surface Fas receptor (CD95/Apo-1) and the tumor necrosis factor receptor, and then recruit the adaptor protein FADD and procaspase-8 to form the death-inducing signaling complex (DISC), thereby activating caspase-8 through cleavage, which is the extrinsic or death receptor apoptotic pathway (**Figure 1A**) (23). The activation of caspase-8 for death receptor-mediated apoptosis occurs *via* two different molecular pathways. TNF-α cotreatment with cycloheximide activates caspase-8 by eliminating c-FLIP, whereas TNF-α cotreatment with a Smac mimetic activates caspase-8 by triggering the degradation of cIAP1/2 followed by the release of receptor-interacting serine-threonine kinase 1 (RIPK1/RIP1), thereby inducing apoptosis (24). Furthermore, lysosomal cathepsins might activate caspase-8 in a death receptor- and mitochondria apoptotic pathway-independent manner (25). Besides its apoptotic functions, caspase-8 affects other “programmed” cell death-necroptosis pathways by cleaving the death domain kinase RIP1 (RIPK1)/RIP3 (RIPK3) (**Figure 1B**) (26, 27) to regulate embryonic development (28); thus, caspase-8-mediated apoptosis and necroptosis compete with each other by cleaving different downstream substrates. In addition to being activated in the “programmed” cell death DISC, which is involved in apoptosis and necroptosis, caspase-8 is also activated in inflammasome complexes and controls inflammasome activation (29) to induce microglial activation (30). Accordingly, caspase-8 deficiency in humans causes defects in the activation of lymphocytes and natural killer cells, indicating the critical role of caspase-8 in the immune activation of lymphocytes (31, 32). Moreover, the DISC components FADD and caspase-8 regulate NLRP3 inflammasome priming and activation (**Figure 1C**) (29, 33, 34), suggesting that DISC and inflammasome complexes might intercross with each other.

## Caspase-8 Activation by Bortezomib and Lenalidomide in Myeloma Cells

Bortezomib (PS-341), a reversible proteasome inhibitor, was approved by the FDA for the treatment of multiple myeloma in 2003 (35). Similarly, lenalidomide and pomalidomide have been approved by the FDA, in 2005 and 2013, respectively, for the treatment of multiple myeloma (36). Since then, the bortezomib–lenalidomide combination has been used as the first-line treatment for multiple myeloma (37–39); however, there is a therapeutic paradox associated with lenalidomide and bortezomib treatment in myeloma (17). Bortezomib could potentiate the anti-myeloma effect of lenalidomide by stabilizing CRBN after short-term treatment in myeloma cells (13). Furthermore, although bortezomib blocks the degradation of IKZF1/3, ubiquitinated IKZF1/3 might not have transcriptional activity, and thus could mediate the anti-myeloma effect of lenalidomide (17).

Bortezomib activates caspase-8 in myeloma cells, thereby inducing apoptosis of myeloma cells (4). Bortezomib induced oxidative stress in human hepatoma HepG2 cells and subsequently activated JNK, thereby activating caspase-8 by increasing the FasL expression (40), which is dependent on the death receptor. Moreover, bortezomib could induce autophagy and then activate caspase-8 in a death receptor-independent manner (41). Interestingly, the relationship between autophagy and caspase-8 was first identified in T lymphocytes (42), and low activated caspase-8 was required for T-cell proliferation (**Figure 1D**) (42, 43).

Lenalidomide recruits the neo-substrate IKZF1/3 onto CRBN and promotes ubiquitination and subsequent degradation by the CRL4^CRBN^ E3 ligase, thereby exhibiting an anti-myeloma effect (7–9). Although the anti-proliferative effect constitutes the major anti-myeloma effect of lenalidomide, a caspase-8 fluorometric assay indicated that lenalidomide and pomalidomide could activate caspase-8 (3, 44, 45). Nonetheless, active caspase-8-mediated apoptosis of myeloma cells might be an edge effect of lenalidomide, because cleaved caspase-8 was not detected after a lenalidomide challenge in myeloma cells (5, 44, 45), which indicated that lenalidomide might promote the activities of caspase-8 to low levels that are non-lethal for myeloma cells.

Caspase-8 might participate in different multiprotein platforms to regulate apoptosis, necroptosis, pyroptosis, and proliferation (**Figure 1**) (46). The above mentioned cellular responses were regulated by the enzymatic and nonenzymatic activities of caspase-8, which depended on different cell types and drug-mediated induction. To dissect the enzymatic and nonenzymatic activities of caspase-8, conditional *caspase-8* knockout mice and derived cells were employed to investigate the possible functions of caspase-8 in cellular responses (47, 48). Necroptosis in *caspase-8*-deficient mice was rescued by the deletion of Rip3 (49, 50) or Cyld (51), suggesting that caspase-8 exerted an antinecroptotic effect by cleaving RIP3 or CYLD. Mechanistically, the mixed-lineage kinase domain-like protein (MLKL) was phosphorylated by RIP3 and mediated TNF-α-induced necroptosis (52–54). Embryos of enzymatically inactive caspase-8 (C362S) mice died after necroptosis and pyroptosis, which demonstrated that the enzymatic activity of caspase-8 was crucial for suppressing necroptosis and pyroptosis and further underscored the suppressive effect of caspase-8-mediated apoptosis on necroptosis and pyroptosis (46, 55, 56). The *Mlkl* deficiency rescued the necroptosis of caspase-8 (C362S) mice but resulted inpyroptosis (46), which indicated that caspase-8-mediated necroptosis might inhibit pyroptosis, thereby controlling normal embryonic development and adult tissue homeostasis (57).

**Figure 2:**
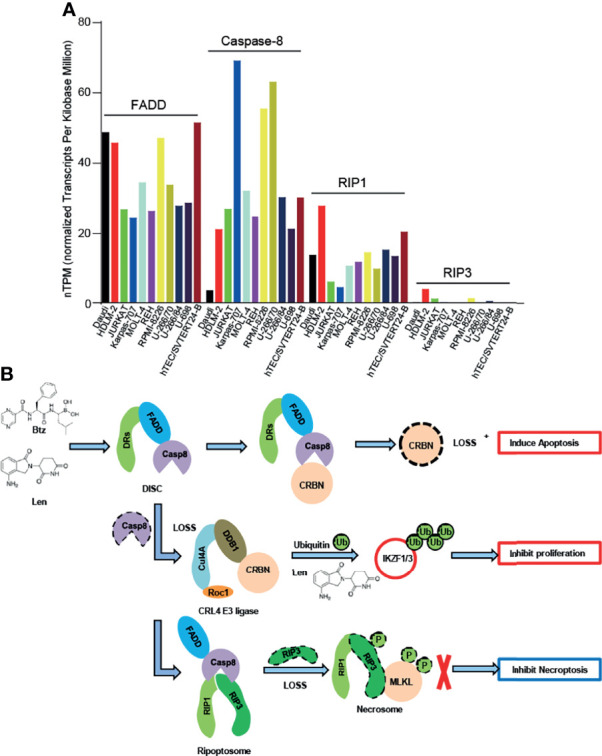
Caspase-8-regulated apoptosis and proliferation of myeloma cells. **(A)** *FADD*, *Caspase-8*, *RIP1*, and *RIP3*mRNA expression in lymphoid cell lines. Data were obtained from the Human Protein Atlas (https://www.proteinatlas.org/). **(B)** Bortezomib (Btz) and lenalidomide (Len) could activate caspase-8 and then cleave CRBN, thereby inducing apoptosis in myeloma cells. When caspase-8 decreased after bortezomib and/or lenalidomide treatment, the CRL4CRBN E3 ligase promoted ubiquitination-mediated degradation of IKZF1/3, thereby inhibiting the proliferation of myeloma cells. Due to the low expression of RIP3 in myeloma cells, necrosomes could not be formed and, therefore, necroptosis was inhibited.

The relatively high level of expression of caspase-8 in lenalidomide-resistant RPMI-8226 cells suggested that the expression of caspase-8 might affect lenalidomide sensitivity in myeloma cells (**Figure 2A**); however, this hypothesis has not been investigated yet. The relatively low expression or loss of *RIP3* expression in myeloma cells (**Figure 2A**) suggests that necroptosis and pyroptosis might be harder to induce than apoptosis, although this needs to be further investigated. Therefore, bortezomib and lenalidomide might not induce necroptosis and pyroptosis in myeloma cells (**Figure 2A**).

With continuous exposure to bortezomib and lenalidomide, bortezomib- and lenalidomide-resistant myeloma cells could be generated from their parental sensitive myeloma cells (58, 59). An examination of the relative expression levels of *FADD*, *caspase-8*, *RIP1*, and *RIP3* in the lenalidomide- and bortezomib-resistant myeloma cells and their parental myeloma cells might help identify a possible programmed cell death pathway to overcome drug resistance.

## Caspase-8-Induced Cleavage of CRBN in Myeloma Cells

CRBN is a lenalidomide-binding protein that mediates the anti-myeloma effect of lenalidomide (8, 9, 60). However, CRBN was not required for bortezomib-induced apoptosis in multiple myeloma (10). Nonetheless, bortezomib could potentiate the anti-myeloma effect of lenalidomide (16, 59), suggesting that bortezomib may regulate the CRBN–lenalidomide–IKZF1/3 signaling pathway. Accordingly, short-term bortezomib treatment inhibited the ubiquitination-mediated degradation of CRBN in myeloma cells, thereby potentiating the anti-myeloma effect of lenalidomide (13). Interestingly, long-term bortezomib treatment blocked the lenalidomide-induced degradation of neo-substrate IKZF1/3 (61) and induced CRBN cleavage (6), which might attenuate the anti-myeloma effect of lenalidomide (**Figure 2B**). Furthermore, as lenalidomide induced low activities of caspase-8, we demonstrated that lenalidomide could regulate the protein level of CRBN by inducing low expression of active caspase-8 (5). Therefore, these studies suggested that short-term bortezomib treatment potentiated the benefits of lenalidomide-based therapy of myeloma patients, whereas long-term bortezomib treatment attenuated the anti-myeloma effect of lenalidomide.

Caspase-8 regulated NF-κB activation in an enzymatic activity-dependent or an enzymatic activity-independent manner, which might be a cell type-or drug treatment-specific effect (47, 62, 63). The enzymatic activity of caspase-8 induced the release of DED-prodomain fragments, thereby activating NF-κB signaling in mouse embryonic fibroblasts following poly(I:C) stimulation (64). However, caspase-8 bound to TRAF2 and FLASH, and thus mediated TNF-α-induced NF-κB activation in NIH3T3, HeLa, HEK293, and T cells, in an enzymatic activity-independent manner (65). Interestingly, the protein levels of CRBN and its neo-substrate IKZF1/3 were unaffected in TRAF2 knockout myeloma cells, although these TRAF2 knockout cells were resistant to both lenalidomide and pomalidomide (66), which suggested that the nonubiquitin functions of CRBN were crucial for cellular responses, such as proliferation. This hypothesis was underscored by the observations that CRBN inhibited NF-κB activation by directly binding to TRAF6 (67, 68), thereby exhibiting nonubiquitin-mediated functions (69). Both caspase-8 and CRBN contributed to NF-κB activation, which further indicated an overlap between caspase-8 and CRBN activities. However, the subsequent functions of CRBN cleavage by caspase-8 on NF-κB activation and cellular responses have not been investigated.

## Caspase-8-Mediated Regulation of Lymphoid Cell Proliferation

FADD and caspase-8 are essential for the cell-cycle progression of T cells, suggesting that caspase-8 also regulated cell proliferation (**Figure 1D**) (43). In line with the above statement, low caspase-8 activities were observed during normal T cell clonal expansion (42). Mechanistically, autophagy was activated in T cells after mitogenic ligand challenge and subsequent recruitment of caspase-8 to FADD : Atg5-Atg12 multiprotein platforms, thereby promoting T cell proliferation (**Figure 1D**) (42). In caspase-8-deficient T cells, autophagy was hyperactivated (42), suggesting that physiologically low activated caspase-8 suppressed the activation of autophagy. Conversely, autophagy could activate caspase-8 (41), indicating the presence of a feedback loop in caspase-8 and autophagy activation in T cell expansion. Notably, the activation of caspase-8 in proliferative T cells did not cause T cell death (43, 70), indicating that caspase-8 was less activated during T cell proliferation. Thus, the sublethal activation of caspase-8 regulated cell-cycle progression, whereas the elevation of active caspase-8 expression following the blocking of protein degradation by bortezomib would be lethal for cells. Taken together, these data suggested that caspase-8 was required for lymphocyte development and activation. Given that myeloma is a cancer of plasma cells, which are differentiated lymphocytes, the possible functions of caspase-8 in the proliferation of myeloma cells need to be further investigated.

## Future Perspectives

Caspase-8 plays a central role in “programmed” cell death, such as apoptosis, necroptosis, and pyroptosis, which is cell type- and drug treatment-specific (46, 71). Furthermore, caspase-8 could enhance or attenuate the tumor malignancy, which is also a cell type- and drug treatment-specific function. Bortezomib and lenalidomide activate caspase-8 and then cleave CRBN, thereby decreasing the sensitivity of the combination-treatment regimen of bortezomib and lenalidomide (5, 6). The inhibition or genetic depletion of caspase-8 then stabilized CRBN, thereby promoting the antiproliferative effect of bortezomib and lenalidomide (6). The necrosome comprises RIP1, RIP3, and MLKL (72, 73). However, myeloma cells might not contain sufficient RIP3 (**Figure 2A**), suggesting that caspase-8 inhibition and genetic depletion blocked not only apoptosis but also necroptosis and pyroptosis in myeloma cells (**Figure 2B**). Given that lenalidomide exerts an anti-myeloma effect by suppressing the proliferation of myeloma cells (8, 9), a physiologically low threshold concentration of active caspase-8 cleaves CRBN and attenuates the anti-myeloma effect of bortezomib and lenalidomide, which is detrimental for myeloma patients. Clinically, it is possible to block the activity of caspase-8 in myeloma patients, thereby enhancing the anti-myeloma effect of lenalidomide; however, no clinical study has evaluated the beneficial effect of caspase-8 inhibition in myeloma patients. Therefore, the inhibition or genetic depletion of caspase-8 might be beneficial for myeloma patients receiving lenalidomide-based therapy.

## Author Contributions

The author confirms being the sole contributor of this work and has approved it for publication.

## Funding

This work was supported by the National Natural Science Foundation of China (32170975).

## Conflict of Interest

The author declares that the research was conducted in the absence of any commercial or financial relationships that could be construed as a potential conflict of interest.

## Publisher’s Note

All claims expressed in this article are solely those of the authors and do not necessarily represent those of their affiliated organizations, or those of the publisher, the editors and the reviewers. Any product that may be evaluated in this article, or claim that may be made by its manufacturer, is not guaranteed or endorsed by the publisher.
